# G protein-coupled receptors in cochlea: Potential therapeutic targets for hearing loss

**DOI:** 10.3389/fnmol.2022.1028125

**Published:** 2022-10-12

**Authors:** Xiangyu Ma, Jiamin Guo, Yaoyang Fu, Cangsong Shen, Pei Jiang, Yuan Zhang, Lei Zhang, Yafeng Yu, Jiangang Fan, Renjie Chai

**Affiliations:** ^1^State Key Laboratory of Bioelectronics, Jiangsu Province High-Tech Key Laboratory for Bio-Medical Research, Department of Otolaryngology Head and Neck Surgery, Zhongda Hospital, School of Life Sciences and Technology, Advanced Institute for Life and Health, Southeast University, Nanjing, China; ^2^Department of Psychiatry, The First Affiliated Hospital, Zhejiang University School of Medicine, Hangzhou, China; ^3^College of Life Science and Technology, Huazhong University of Science and Technology, Wuhan, China; ^4^Jiangsu Provincial Key Medical Discipline (Laboratory), Department of Otolaryngology Head and Neck Surgery, Affiliated Drum Tower Hospital of Nanjing University Medical School, Nanjing, China; ^5^Research Institute of Otolaryngology, Nanjing, China; ^6^Department of Otorhinolaryngology, Head and Neck Surgery, The Second Hospital of Anhui Medical University, Hefei, China; ^7^First Affiliated Hospital of Soochow University, Soochow, China; ^8^Department of Otolaryngology Head and Neck Surgery, Sichuan Provincial People’s Hospital, University of Electronic Science and Technology of China, Chengdu, China; ^9^Co-Innovation Center of Neuroregeneration, Nantong University, Nantong, China; ^10^Institute for Stem Cell and Regeneration, Chinese Academy of Sciences, Beijing, China; ^11^Beijing Key Laboratory of Neural Regeneration and Repair, Capital Medical University, Beijing, China

**Keywords:** hearing loss, G protein-coupled receptors, cochlea, drug therapy, gene therapy

## Abstract

The prevalence of hearing loss-related diseases caused by different factors is increasing worldwide year by year. Currently, however, the patient’s hearing loss has not been effectively improved. Therefore, there is an urgent need to adopt new treatment measures and treatment techniques to help improve the therapeutic effect of hearing loss. G protein-coupled receptors (GPCRs), as crucial cell surface receptors, can widely participate in different physiological and pathological processes, particularly play an essential role in many disease occurrences and be served as promising therapeutic targets. However, no specific drugs on the market have been found to target the GPCRs of the cochlea. Interestingly, many recent studies have demonstrated that GPCRs can participate in various pathogenic process related to hearing loss in the cochlea including heredity, noise, ototoxic drugs, cochlear structure, and so on. In this review, we comprehensively summarize the functions of 53 GPCRs known in the cochlea and their relationships with hearing loss, and highlight the recent advances of new techniques used in cochlear study including cryo-EM, AI, GPCR drug screening, gene therapy vectors, and CRISPR editing technology, as well as discuss in depth the future direction of novel GPCR-based drug development and gene therapy for cochlear hearing loss. Collectively, this review is to facilitate basic and (pre-) clinical research in this area, and provide beneficial help for emerging GPCR-based cochlear therapies.

## Introduction

**FIGURE 1:**
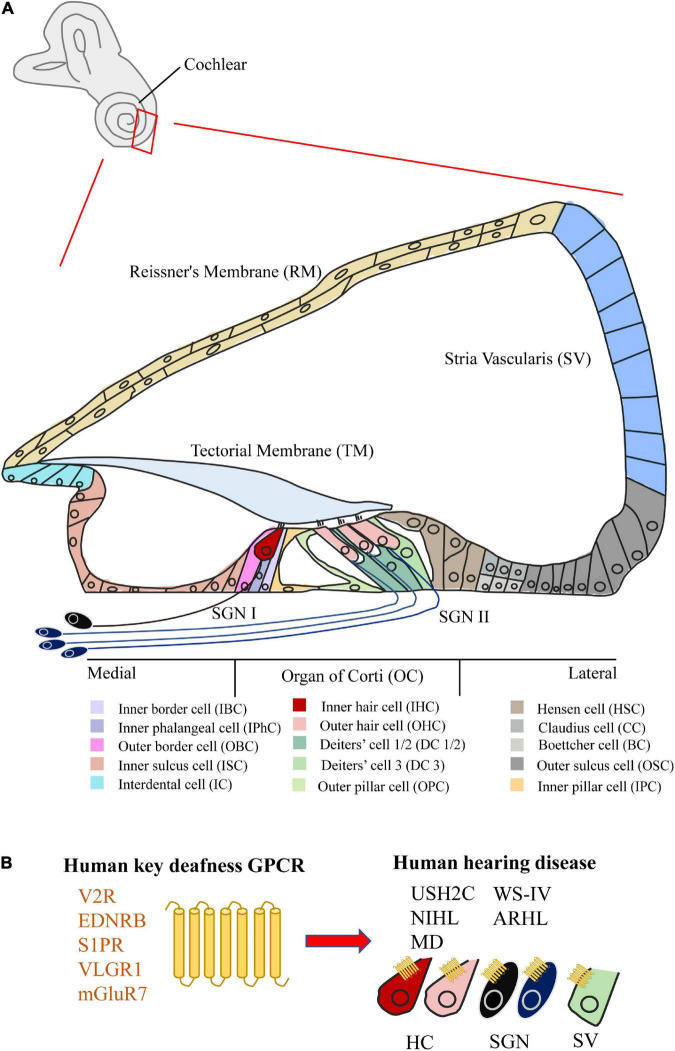
Structure of the cochlea and the GPCR related to human hearing disease. **(A)** Schematic of the mammalian mature cochlea (cross-section). The cochlea can be divided into five parts, including Organ of Corti (OC), Stria Vascularis (SV), Reissner’s Membrane (RM), Spiral Ganglion Neurons (SGNs), Bony Labyrinth (BL) (not shown), and Mesenchymal Cell (MC) (not shown). Among them, two kinds of hair cell (HC) and various kinds of supporting cell (SC) can be subdivided in OC. **(B)** The summary of five key GPCRs (V2R, EDNRB, S1PR, VLGR1, and mGluR7) in cochlea related to human hearing disease (USH2C, WS-IV, NIHL, ARHL, and MD).

Hearing disease currently affects nearly 1.5 billion people worldwide.^1^ Most hearing loss is mainly occurred in the cochlea of the inner ear. The cochlea can be divided into five parts: Organ of Corti (OC), Stria Vascularis (SV), Reissner’s Membrane (RM), Mesenchymal Cell (MC), Bony Labyrinth (BL), and Spiral Ganglion Neurons (SGNs) (Figure 1A; van der Valk et al., 2021; Kelley, 2022). Among them, the OC is the main organ of sound perception in the cochlea, particularly the hair cells (HCs) and various supporting cells (SCs) of the OC are essential for hearing (Wagner and Shin, 2019; Driver and Kelley, 2020), but cell abnormalities in other areas of the cochlea can also cause hearing damage (Jang et al., 2022). Hearing damage can be caused by a variety of factors, including heredity, noise, ototoxic drugs, damage to the cochlear environment and structure. However, current treatment options (cochlear implants and hearing aids) mainly depend on the capacity of residual HCs and SGNs to improve the patient’s hearing level to a certain extent (Wolf et al., 2022).

G protein-coupled receptors (GPCRs) with seven transmembrane domains are the largest superfamily of mammalian cell surface receptors, which have more than 800 members (Foord et al., 2005; Wingler and Lefkowitz, 2020). GPCRs govern a wide range of physiological processes, such as hormone release, neurotransmitter transmission, and immune responses, mainly through the recognition and activation of heterotrimeric G proteins (Gα, Gβ, and Gγ) by binding to a variety of ligands (proteins, peptides, and lipids, etc.), as well as GPCRs phosphorylated by G protein-coupled receptor kinases (GRKs) to recruit β-arrestins and internalize and inactivate GPCRs (Wang et al., 2018; Insel et al., 2019). Based on structural similarity, GPCRs in humans are divided into five major families: Rhodopsin receptors (Class A), Secretin receptors (Class B1), Adhesion receptors (Class B2), Glutamate receptors (Class C), and Frizz/Taste 2 (Class F) (Kochman, 2014; Hauser et al., 2017). Currently, more than 100 GPCRs could be regarded as therapeutic targets, especially more than 30% of marketed drugs have been designed for GPCRs (Hauser et al., 2017).

Compared with extensive studies on GPCRs in mental illness (Pasquini et al., 2022) and cancer (Chaudhary and Kim, 2021), the function studies of GPCRs in the cochlea are very limited, scattered, and unsystematic. In particular, there are still no specific drugs on the market targeting the GPCR of the cochlea. With the in-depth study of GPCRs in the cochlea, we believe that more GPCR functions in the cochlea will be revealed, and more drugs and treatment programs targeting cochlear GPCRs will be discovered. In this review, we therefore characterized the distribution and function of 53 GPCRs expressed in the cochlea, as well as their relationships with hearing loss. Notably, five GPCRs (V2R, EDNRB, S1PR, VLGR1, and mGluR7) of them in the cochlea have been reported to be directly associated with human hearing disorders (Figure 1B). We also summarize the new advances in cochlear research techniques, and suggest the future direction of novel GPCR-based drug development and gene therapy for cochlear hearing loss.

## Roles of class A G protein-coupled receptors in cochlea

### Vasopressin and oxytocin receptors

Vasopressin type 2 receptor (V2R) is primarily expressed in the kidney and participates in controlling water homeostasis (Kim et al., 2021; Zhou et al., 2021). V2R is activated by arginine vasopressin (AVP), which in turn induces the buildup of downstream cAMP (Wang et al., 2021). Numerous V2R-related human diseases have been identified, including nephrotic syndrome of inappropriate diuresis (NSIAD), X-linked congenital nephrogenic diabetes insipidus (NDI), and hyponatremia (Makita et al., 2020). V2R-related antagonists have also been extensively studied, such as Tolvaptan (de la Nuez Veulens et al., 2022).

In the cochlea, V2R is mainly expressed in HCs, SGNs, and SVs (Takumida et al., 2012). V2R is thought to play a role in endolymphatic hydrops (EH). EH is caused by an imbalance in the volume of endolymph and is thought to be associated with the pathology of Menière’s disease (MD) that is a kind of hearing loss’s inner ear disease (Zou et al., 2019; Wang et al., 2022). EH can be also inhibited *via* reducing the expression of V2R. The degree of cochlear hydrops can be alleviated by applying the V2R antagonist (OPC-41061/Tolvaptan) (Egami et al., 2016). Additionally, the expression level of V2R in the cochlea can be significantly inhibited by vincamine, thereby reducing EH and regulating hearing (Li et al., 2018). However, studies found that EH is significantly attenuated by electroacupuncture (EA), but V2R is up-regulated (Jiang L. et al., 2019; Jiang L. Y. et al., 2019). Therefore, it is still needed to validate the function of V2R in the regulation of EH.

### Endothelin receptors

Endothelin receptor B (EDNRB) is activated by endothelins (ETs), and three ETs (ET-1, ET-2, and ET-3) have equal affinity for EDNRB (Shihoya et al., 2016; Izume et al., 2020). EDNRB is widely expressed in circulatory organs including vascular endothelium, brain, and intestine, especially endothelin receptor antagonists have been used to treat circulatory system diseases (Bondurand et al., 2018; Shihoya et al., 2018). Heterozygous and homozygous mutations in EDNRB and ET-3 are found in patients of Waardenburg-Shah syndrome (WS-IV) that is one kind of syndromes with genetic hearing loss (GHL) (Huang et al., 2021). Homozygous mutations of the *EDNRB* gene can be also identified in Moroccan deaf patients (AitRaise et al., 2022).

EDNRB is expressed in SV’s melanocytes and the SGN of the cochlea, which is important for postnatal hearing development (Ida-Eto et al., 2011; Renauld et al., 2021). In mouse models, both *EDNRB* spontaneously mutated and *EDNRB* homozygous knockout (*EDNRB*^–/–^) mice developed severe congenital deafness (Gariepy et al., 1996; Matsushima et al., 2002). In the case of *EDNRB*^–/–^, melanocytes in the SV of the cochlea are defective and the SGN undergoes postnatal degeneration (Ida-Eto et al., 2011). The introduction of human *DBH-EDNRB* transgene can restore the SGN of *EDNRB*^–/–^ mice to a certain extent and improve the hearing levels, but the defect of melanocytes does not change (Ida-Eto et al., 2011). Therefore, targeted modulation of EDNRB expression in SGN might be a new strategy to treat congenital hearing loss patients with WS-IV.

### Glycoprotein hormone receptors

Thyrotropin receptor (TSHR) can be activated by thyrotropin (TSH), which then stimulates thyroid hormone production through the G_*s*_ and G_*q*_ signaling pathways (Tuncel, 2017; Vieira et al., 2022). Abnormalities in TSHR can lead to autoimmune diseases such as hypothyroidism and hyperthyroidism. Recent studies have elucidated the structures of the activated and inactivated states of TSHR with different types of antibodies, which provides a structural basis for subsequent antibody drug and small molecule drug discovery (Duan et al., 2022; Faust et al., 2022).

In a mouse model, *TSHR^hyt/hyt^* mutant mice express the gene encoding the *TSHR* with a point mutation in the highly conserved transmembrane structure, rendering TSHR unable to bind with TSH (Gu et al., 1995). The autosomal recessive *TSHR^hyt/hyt^* mutant mice develop severe hearing loss, and outer hair cells (OHCs) of the cochlea exhibit developmental defects and loss of functional integrity (O’Malley et al., 1995; Li et al., 1999; Song et al., 2006). Of note, the exact role of TSHR in the cochlea remains currently in a stagnant state.

### Lysophospholipid (S1P) receptors

Sphingosine-1-phosphate receptors (S1PR1 to S1PR5) can be involved in the regulation of immune and vascular systems after activation by sphingosine-1-phosphate (S1P) (Cartier and Hla, 2019). Three inherited missense mutations (R108P, R108Q, and Y140C) in S1PR2 are found in patients with deaf (Santos-Cortez et al., 2016; Hofrichter et al., 2018). A recent protein structure study revealed that three mutation sites associated with human autosomal recessive hearing loss (ARHL) lead to changes of the protein structure of S1PR2 (Chen et al., 2022). These structural changes affect the binding of S1PR2 to the ligand S1P, to G_13_, suggesting that the S1PR2-G_13_ signaling complex plays a role in maintaining normal hearing function of the cochlea.

*S1PR1-3* was also found to be expressed in the mouse cochlea (Nakayama et al., 2014). Among them, S1PR2 can be specifically expressed in HCs, SCs, SGNs, and SVs (Ingham et al., 2016). In mouse models, both mutation (*S1PR2^stdf/stdf^*) and knockout of S1PR2 (*S1PR2*^–/–^) result in progressive hearing loss, essentially starting at 2–4 weeks postnatally with varying degrees of hearing impairment to complete deafness, which is characterized by the defect of SV at the onset and followed by decreased intracochlear potential (EP) and subsequent loss of HCs and SGNs (Herr et al., 2007, 2016; Kono et al., 2007; Ingham et al., 2016). Remarkably, mice lacking the *S1PR3* did not develop a hearing impairment phenotype (Kono et al., 2007). S1PR2 is also a potential target to protect hearing loss by preventing the ototoxic drugs induced apoptosis of HC and SGN. Administration of an S1PR2 antagonist (JTE013) resulted in the increase of gentamicin ototoxicity (Nakayama et al., 2014), whereas administration of an S1PR2 agonist (CYM-5478) reduced the cisplatin ototoxicity by reducing ROS accumulation (Wang et al., 2020). Of note, antagonists of S1PR1 and S1PR3 failed to increase gentamicin ototoxicity (Nakayama et al., 2014). Overall, S1PR2 plays a key role in maintaining hearing function and inhibiting damage caused by ototoxicity, particularly it is worthy of in-depth study as an ear protection therapeutic drug target.

### P2Y receptors (purinergic receptors)

P2Y receptor (P2YR), as a GPCR subfamily of eight subunits (P2YR1, 2, 4, 6, 11–14) known, can respond to extracellular nucleotides (Cabou and Martinez, 2022). Among them, P2YR1, 11–13 are activated by ATP/ADP, P2YR4, 6, 14 are activated by UTP/UDP, and P2YR2 receptors are activated by ATP/UTP (Abbracchio et al., 2006; Koles et al., 2019). The expression of six P2YRs (P2YR1, 2, 4, 6, 12, 14) could be detected in the cochlea (Parker et al., 2003; Huang et al., 2010; O’Keeffe et al., 2010; Koles et al., 2019). Before hearing maturation (<P15), five P2YRs except P2YR1 could be detected in both sensory and non-sensory cells in the cochlea. After hearing maturation (>P15), only P2YR12 and P2YR14 could not be detected in HCs. The specific expressions of six P2YRs in the cochlea are shown in Table 1.

**TABLE 1:** Class A GPCRs relevant to cochlea.

GPCR family	Subtypes	Roles in cochlea	Localization	Genetic modulation	References
**Class A GPCRs**					
Vasopressin and oxytocin receptors	V2R	Play a role in endolymphatic hydrops (EH)	HC, SGN, SV	-	Zou et al., 2019; Wang et al., 2022
		Associated with human Menière’s disease (MD) with hearing loss			
Endothelin receptors	EDNRB	Associated with human Waardenburg–Shah syndrome (WS-IV) [a kind of genetic hearing loss (GHL)]	SV’s melanocytes, SGN	*EDNAB^–/–^* mice	Ida-Eto et al., 2011
		Affect hearing function: severe congenital deafness when knockout		*DBH-EDNAB* mice	
		SV’s melanocytes are defective and the SGN undergoes postnatal degeneration when knockout		WS4 mice	Matsushima et al., 2002
Glycoprotein hormone receptors	TSHR	TSHR hyt/hyt mutant mice (autosomal recessive) develop severe hearing loss	-	*TSHR^*hyt*/hyt^* mice	Gu et al., 1995
		Affect OHC development and function			
Lysophospholipid (S1P) receptors	S1PR1	-	Cochlea	-	Nakayama et al., 2014
	S1PR2	Associated with human autosomal recessive hearing loss (ARHL)	HC, SC, SGN, SV	*S1PR2^*stdf*/stdf^*	Ingham et al., 2016
		Lead to progressive hearing loss when knockout or mutated		*S1PR2^–/–^*	Herr et al., 2007
		Affect the development of SV at the onset			
		Inhibit damage caused by ototoxicity			
	S1PR3	No hearing impairment phenotype when knockout	Cochlea	*S1PR3^–/–^* mice	Kono et al., 2007
P2Y receptors (Purinergic receptors)	P2YR1	Play an important role in the burst firing before hearing onset	RM, SC (<P15)	*P2RY1^–/–^* mice	Babola et al., 2020
			OHC, OSC, RM (>P15)	*P2RY1-LacZ* mice	
	P2YR2	Induce the propagation of Ca2+ waves	OHC, IHC, PC, DC, HSC, OSC, SV, RM (<P15)	-	Koles et al., 2019
			OHC, PC, OSC (>P15)		
	P2YR4	Mediate to inhibit Na+ uptake in cochlear RMs	OHC, IHC, SGN, HSC, OSC, SV, RM (<P15)	-	Koles et al., 2019
		Induce the propagation of Ca2+ waves	OHC, IHC, SGN, PC, DC, HSC, OSC, SV, RM (>P15)		
	P2YR6	-	OHC, IHC, SGN, PC, SV, RM (<P15)	-	Koles et al., 2019
			OHC, SGN, PC, SV, RM (>P15)		
	P2YR12	-	OHC, SGN, PC, RM (<P15)	-	Koles et al., 2019
			SGN, RM (>P15)		
	P2YR14	-	OHC, IHC, SGN, OSC (<P15)	-	Koles et al., 2019
			SGN, OSC (>P15)		
Dopamine receptors	DRD1	Activate adenylyl cyclase	OHC, SGN	*DRD1^–/–^* mice	Maison et al., 2012
Chemokine receptors	DRD2	Inhibit adenylyl cyclase	OHC, SGN	*DRD2^–/–^* mice	Maison et al., 2012
	DRD4	Inhibit adenylyl cyclase	SGN	*DRD4^–/–^* mice	Maison et al., 2012
	DRD5	Activate adenylyl cyclase	OHC, SGN	*DRD5^–/–^* mice	Maison et al., 2012
	CXCR4	Regulates cochlear development and stem cell homing	SGN	-	Zhang et al., 2015
	CX3CR1	Regulation of inflammatory response	Macrophages, monocytes	*CX3CR1^–/–^* mice	Zhang et al., 2021
Dopamine receptors	CCR2	Regulating inflammatory response to noise–and drug-induced hearing impairment	Monocytes	*CCR2^–/–^* mice	Hirose and Li, 2019
Cannabinoid receptors	CCR7	Protects against noise-induced auditory cell damage	Monocytes	-	Maeda et al., 2018
Apelin receptors	CB2R	Ototoxicity induced by cisplatin treatment was inhibited, and inflammation and oxidative stress were reduced	OC, SLF, SGN	-	Ghosh et al., 2018
	APJ	Anti-oxidation, prevent cell apoptosis	OHC, IHC	-	Yin et al., 2020
Adenosine receptors	AA1R	Protects the mouse cochlea from noise damage, cisplatin induced ototoxicity, and age-related hearing loss, and reduces the death of auditory cells	SGN, SC, IHC	*AA1R^–/–^* mice	Vlajkovic et al., 2009
	AA2AR	Coupled with G_s_ proteins that promote adenylate cyclase	SGN, OC	*AA2AR^–/–^* mice	Vlajkovic et al., 2017
	AA2BR	Coupled with G_s_ proteins that promote adenylate cyclase	SGN, OC	-	Manalo et al., 2020
	AA3R	Coupled with G_i/o_ proteins inhibit adenylate cyclase activity	SC, IHC	-	Vlajkovic et al., 2007
Class A orphan receptors	GPR26	Deleted as part of a recessive mouse mutant (hb/hb) that exhibits severe hearing impairment	SLF, SGN	*hb/hb* mice	Buniello et al., 2013
				*GPR26^–/–^* mice	Zhang et al., 2011
	LGR4	Involved in the regulation of Wnt/β-catenin activity by playing a permissive role in the Wnt/β-catenin signaling pathway	HC, PC, DC	*LGR4-LacZ* mice	Zak et al., 2016
	LGR5	Regulate cochlear development and promote hair cell regeneration	IPC, SC, DC	*LGR5-eGFP* mice	Cheng et al., 2017
				*LGR5-EGFP-IRES-CreERT2* mice	Zak et al., 2016
	LGR6	Regulation of progenitor cell proliferation	IPC, SC	*LGR6-EGFP-IRES-CreERT2* mice	Zhang Y. et al., 2018

HC, hair cell; SGN, Spiral Ganglion Neuron; SV, Stria Vascularis; SC, supporting cell; RM, Reissner’s Membrane; OHC, outer hair cell; OSC, outer sulcus cell; IHC, inner hair cell; PC, Pillar cell; DC, Deiters’ cell; HSC, Hensen cell; OC, Organ of Corti; IPC, inner Pillar cell; SLF, spiral ligament fibrocytes.

P2YR1, 2, 4 have been functionally studied to some extent in the cochlea. Among them, P2YR1 plays an important role in the burst firing before hearing onset. The maturation of emerging neural circuits is facilitated by spontaneous bursts of electrical activity in the developing nervous system (Blankenship and Feller, 2010). K^+^ release can be triggered when P2RY1 is activated in SCs, thereby activating inner hair cells (IHCs) and SGNs (Babola et al., 2020, 2021). Both pharmacological (MRS2500) inhibition of P2RY1 or *P2RY1* deletion significantly reduced burst firing in SGNs (Babola et al., 2020, 2021). Moreover, P2YR4 mediated the inhibition of Na^+^ uptake in cochlear RMs, possibly in response to noise exposure (Kim et al., 2010). In particular, P2YR2 and P2YR4 in the cochlea can also induce the propagation of Ca^2+^ waves (Piazza et al., 2007).

### Dopamine receptors

G protein-coupled dopamine receptors execute almost all the physiological functions of catecholaminergic neurotransmitter dopamine. This dopamine receptor family includes five GPCR subtypes, and can be divided into two categories: DRD1 (dopamine receptor D1) and DRD5 binding to G proteins and activating adenylyl cyclase; DRD2, DRD3, and DRD4 binding to G proteins and inhibiting adenylyl cyclase (Beaulieu and Gainetdinov, 2011). Transcriptome sequencing of whole cochlear samples from adult mice revealed the presence of DRD1, DRD2, DRD4, and DRD5 transcripts, but not DRD3 mRNA. DRD1, DRD5, and DRD2 receptors were expressed in OHCs and SGNs, but DRD4 receptors were expressed only in SGNs (Maison et al., 2012).

There is no consensus on which receptors can mediate the hearing (Meredith and Rennie, 2021), but it is widely accepted that activated dopamine receptors can decrease the excitotoxicity of IHC synapses through the effect of dopamine on afferents (Oestreicher et al., 1997). Current biochemical and pharmacological evidence suggests that dopamine release from lateral cochlear efferent neurons can inhibit the cochlear nerve fiber activity (Gaborjan et al., 1999; Ruel et al., 2001). Studies have also shown that the administration of dopamine and dopaminergic agonists may reduce the action potentials’ firing rate in frog semicircular canal afferents (Andrianov et al., 2009). In guinea pigs and rats, dopamine reduced the rate of action potential firing from cochlear auditory afferents (Oestreicher et al., 1997; Wu et al., 2020). Exposure to sound also raise dopamine in mouse efferent neurons, revealing that dopamine has very vital neuroprotective effect (Maison et al., 2012; Wu et al., 2020). When the DRD1, DRD2, DRD4, and DRD5 dopamine receptor knockout mice were respectively exposed to noise, all four mutants demonstrated increased vulnerability (Maison et al., 2012). These studies not also support the role of dopaminergic signaling in the HC system of different species, but reveal its potential application value in hearing protection.

### Chemokine receptors

Chemokines regulate cell migration and proliferation, as well as immune and inflammatory responses. Twenty chemokine receptors have been identified, including four subfamilies (Sanchez et al., 2019). Chemokine receptors and chemokines can participate in various physiological and pathological processes, including cancer cell growth (Smith et al., 2004) and metastasis (Zlotnik et al., 2011), angiogenesis (Keeley et al., 2010; Lin et al., 2015), and immune responses of patient prognosis (He et al., 2022). Chemokine receptors reported in the cochlea include CXCR4, CX3CR1, CCR2, and CCR7.

CXCR4 and its ligand CXC chemokine ligand 12 (CXCL12, also called as stromal cell-derived factor-1) involve in regulating neural stem cell migration, differentiation and maturation (Zhang et al., 2015), vertebrate embryogenesis (Peyvandi et al., 2018b). In the cochlea, CXCR4 protein is mainly expressed in SGNs. CXCR4/CXCL12 participates in cochlear development in neonatal mice and rats (Zhang et al., 2015, 2016), and stem cell homing in noise-induced injury areas in adult rats (Zhang et al., 2014; Peyvandi et al., 2018a). CX3CR1, as a receptor for the chemokine Fractalkine, is found to express in NK cells, macrophages, monocytes, microglia, and partly T cells (Jung et al., 2000). CX3CR1 is also expressed in macrophages and monocytes of the mouse cochlea (Claussen et al., 2022). In response to the transformation of monocytes and migration of macrophages in hearing damage caused by noise stimulation (Shin et al., 2022a,b), CX3CR1 regulates the inflammatory response caused by cochlear injury (Zhang et al., 2021). CX3CR1-deficient cochlear macrophages can also aggravate the ototoxicity of kanamycin (Sato et al., 2010). Remarkably, CCR2 and CCR7 may be also involved in regulating inflammatory response in hearing impairment induced by noise and drugs (Sautter et al., 2006; Maeda et al., 2018; Hirose and Li, 2019). Therefore, chemokine receptors and chemokines play important roles in cochlear development, stem cell homing and immune response after hearing damage, suggesting them with a potential to repair hearing damage and protect nerves.

### Cannabinoid receptors

Cannabinoid 2 receptors (CB2Rs), one type of cannabinoid receptor, are found in peripheral tissues of immunological origin (Munro et al., 1993; Brown et al., 2002) and are distributed in different brain regions (Ishiguro et al., 2022). CB2Rs are post-synaptically expressed and up-regulated in response to injury and inflammation (Ishiguro et al., 2022). CB2Rs are mainly distributed in OC, spiral ligament and SGN cells in the cochlea of rats and mice (Kim et al., 2014; Kaur et al., 2016; Ghosh et al., 2018). CB2Rs can protect the cochlea and reduce ototoxicity, inflammation and oxidative stress with cisplatin treatment in rats (Vlajkovic et al., 2006; Dhukhwa et al., 2021). The effect of CB2Rs on preventing cisplatin induced hearing loss was blocked by injection of the antagonist AM630, but HC loss was reduced by injection of JWH105 (one agonist of CB2R). Of note, after knock-down of CB2Rs by siRNA, the cochlea is more sensitive to cisplatin induced hearing loss (Ghosh et al., 2018). Therefore, CB2Rs may be an important therapeutic target against ototoxicity.

### Apelin receptors

Apelin receptor (APJ) and its ligand Apelin are key participators involved in the regulation of oxidative stress. Among various subtypes of Apelin, Apelin-13 has the strongest biological activity (Niknazar et al., 2019). APJ/Apelin is widely expressed in the heart, brain, kidney, stomach and intestines (Fournel et al., 2017; Lv et al., 2017), and has antioxidant and apoptotic effects in distinct cell types (Bircan et al., 2016; Aminyavari et al., 2019). Apelin attenuates DNA damage caused by ROS accumulation in cisplatin-induced myocardial toxicity (Zhang P. et al., 2017). Apelin-13 protects cardiomyocytes by reducing oxidative damage in a rat model of myocardial infarction (Azizi et al., 2013).

Both APJ and Apelin are expressed in mouse cochlear HCs and HEI-OC1 cells, and the expression of APJ in OHCs is significantly higher than that in IHCs (Yin et al., 2020). Cisplatin can down-regulate the expression of Apelin in HCs and HEI-OC1 cells, and treatment with Apelin in advance can improve the survival rate of HEI-OC1 cells under cischloride ototoxicity and alleviate the damage of cochlear mitochondrial membrane potential by ROS (Yin et al., 2020). In addition, noise-induced oxidative stress and DPOAE response were significantly altered and inhibited by Apelin-13 pretreatment (Khoshsirat et al., 2021).

### Adenosine receptors

This adenosine receptor family includes four GPCRs, designated as A1, A2A, A2B, and A3. Adenosine A1 receptor (AA1R) and adenosine A3 receptor (AA3R) coupled with G_*i/o*_ proteins to inhibit adenylate cyclase activity, whereas adenosine A2A receptor (AA2AR) and adenosine A2B receptor (AA2BR) coupled with G_*s*_ proteins to activate adenylate cyclase (Vlajkovic et al., 2007). Together, the adenosine receptor family and its signaling molecules regulate cellular activity in peripheral organs.

The distribution of four adenosine receptors in the cochlea is diverse (Vlajkovic et al., 2009; Manalo et al., 2020). AA1R is distributed in SGNs, and in SCs and IHCs of the OC, but AA2AR and AA2BR localize to SGNs, OC, and cochlear vessels. AA3R is mainly expressed in SCs and inner HCs of the OC. The balance between AA1R and AA2AR determines the cochlear response to oxidative stress. AA1R can protect the cochlea of mice from noise injury, cisplatin-induced ototoxicity and age-related hearing loss (Vlajkovic et al., 2011, 2017; Sheth et al., 2019). Similar results have been reported in rat, chinchilla and guinea pig (Ramkumar et al., 1994, 2004; Tabuchi et al., 2012). Administration of AA1R probiotics R-PIA and ADAC was more significant in inhibiting cisplatin-induced ototoxicity (Vlajkovic et al., 2010; Kaur et al., 2016), and the protective effect of R-PIA was inhibited by combined use of AA1R antagonist DPCPX (Whitworth et al., 2004). In contrast, AA2AR and AA2BR play a negative regulatory role in hearing loss, with cochlear protection achieved by the use of the inhibitor (istradefylline) (Han et al., 2019; Manalo et al., 2020; Shin et al., 2021).

### Class A orphan receptors

GPR26, one class of orphan receptors for Class A, is mainly expressed in the brain and attracted attention due to its role in central nervous system diseases (Alavi et al., 2018; Watkins and Orlandi, 2020). *GPR26* is deleted along with two other genes (*CPMX2* and *CHST15*) in recessive mouse mutant mice (*hb/hb*) that exhibit severe hearing impairment (Buniello et al., 2013). Symptoms of anxiety and depression were presented in *GPR26* knockout mice, but no hearing function was reported (Zhang et al., 2011). In the mouse cochlea, GPR26 expression was detected in spiral ligament fibrocytes (SLF) and SGNs (Buniello et al., 2013), but *hb/hb* mutant mice in the cochlea without GPR26 expression, indicating that it is worth using the *GPR26*^–/–^ mice to examine the role of GPR26 in the cochlea.

Another class of orphan receptors for Class A is one member of the leucine-rich repeat-containing G-protein-coupled receptors (LGRs) family. LGR4, LGR5, and LGR6 could be expressed in the cochlea (Zak et al., 2016; Zhang Y. et al., 2018; Smith-Cortinez et al., 2021). LGR5 is considered as a marker of cochlear stem cells and participates in the development of auditory HCs (Smith-Cortinez et al., 2021). LGR5 regulates cochlear development by enhancing the Wnt/β-catenin signaling pathway (Cheng et al., 2017; McLean et al., 2017), especially LGR5-positive SCs have the potential to transdifferentiate into HCs, suggesting that it may be acted as a therapeutic target for hearing loss (Cox et al., 2014; Zhang S. et al., 2017; Smith-Cortinez et al., 2021; Ma et al., 2022). LGR5-deficient mice produce additional HCs, and LGR4-deficient mice show similar results (Zak et al., 2016). LGR6^+^ cells, a subtype of LGR5^+^ progenitor cells, also regulate progenitor cell proliferation and HC production (Zhang Y. et al., 2018).

## Roles of class B1 G protein-coupled receptors in cochlea

### Vasoactive intestinal peptide and pituitary adenylate cyclase-activating peptide receptors

Vasoactive intestinal peptide (VIP) and pituitary adenylate cyclase-activating peptide (PACAP) receptors include VPAC1R, VPAC2R, and PAC1R, which are activated by VIP and PACAP (Hollenstein et al., 2014; Langer et al., 2022). Among them, unlike VPAC1R and VPAC2R, PAC1R can specifically binds to PACAP but has a lower affinity toward VIP (Vaudry et al., 2009). These two neuropeptides are widely distributed and involved in development, anti-apoptosis, and neuroprotection together with their receptors (Langer et al., 2022). In the cochlea, VIP and VIP receptors are mainly expressed in SGNs (Kitanishi et al., 1998). In addition, the levels of VIP and VPAC1R were down-regulated in the cochlea of chronic alcoholic rats, implying that they might act as neurotransmitters (Feng and Liu, 2015). However, there are still few studies on VIP and VIP receptors, and their specific mechanisms of action in the cochlea need to be further studied.

PACAP and PAC1R are expressed in HCs, SCs, SGNs, afferent, and efferent nerve fibers, and stria vascularis of the cochlea (Abu-Hamdan et al., 2006; Drescher et al., 2006; Ruel et al., 2021). Endogenous PACAP plays important roles in protection against noise-induced hearing loss (NIHL) (Ruel et al., 2021), maintenance of hearing during aging in mice (Fulop et al., 2019) and against oxidative stress-induced apoptosis (Racz et al., 2010). Current functional studies focused on the role of PACAP in the cochlea as well as the PAC1R in the protection of NIHL (Ruel et al., 2021). After noise injury, the *PAC1R^–/–^* knockout mice exhibited a significant increase in hearing threshold, but the humanized mice expressing human PAC1R (TgHPAC1R) showed a relatively small increase in hearing threshold. Taken together, with the establishment of a mouse model corresponding to the PAC1R, other roles of the PAC1R in the cochlea will be gradually uncovered.

### Corticotropin-releasing factor receptors

Corticotropin-releasing factor receptors (CRFRs) in mammals only express CRFR1 and CRFR2, which are activated by corticotropin-releasing factor (CRF). As the main regulator of stress response, they participate in neuroendocrine, metabolism and response to stress (Vetter, 2015; Dedic et al., 2018). Among them, CRFR1 has a higher affinity for CRF than CRFR2 does.

CRFR1 is mainly localized in inner sulcus cells (ISCs), Hensen cells (HSCs), Deiters’ cells (DCs), and border cells (BCs) in the cochlea (Graham and Vetter, 2011), but no expression in HCs and SGNs. CRFR1 plays an important role in maintaining normal auditory function, IHC and HC innervation development. In *CRFR1^–/–^* mice, both ABR thresholds and DP thresholds were elevated, suggesting that elimination of CRFR1 might result in decreased cochlear sensitivity and impaired OHC motility (Graham and Vetter, 2011), as well as defects in IHC, afferent and efferent innervation (Graham and Vetter, 2011).

Likewise, the effects of CRFR2 on the cochlea are diverse. CRFR2 is mainly expressed in ISCs, DCs, inner border cells (IBCs), SGNs, Claudius cells (CCs), and Boettcher cells (BoCs) (Graham et al., 2010), but not in HCs. CRFR2 constitutively modulates hearing sensitivity under normal conditions and performs an important protective function in noise-induced hearing loss. Mice lacking *CRFR2* exhibited significantly lower hearing thresholds under normal conditions, but more severe hearing impairment when exposed to noise (Graham et al., 2010). Interestingly, there was no loss of IHCs or OHCs in the cochlea of *CRFR2^–/–^* mice exposed to moderate ambient noise (Graham et al., 2010). CRFR2 affects cochlear hearing function by acting on glutamatergic transmission, purinergic signaling and activation of Akt/PKB signaling in the cochlea (Graham et al., 2010). Currently CRFR1 and CRFR2 have been considered as promising targets for the treatment of asthma and alcoholism drug therapy (Tantisira et al., 2004; Lowery and Thiele, 2010).

### Calcitonin receptors

Calcitonin Gene-Related Peptide Receptor (CGRPR) is a heterodimeric membrane protein complex composed of receptor activity-modifying protein 1 (RAMP1) and calcitonin receptor-like receptor (CLR) with the ability to bind to CGRP (Liang et al., 2018). As an important sensory neuropeptide, CGRP is widely expressed in the nervous system and exists in two forms, i.e., α-CGRP and β-CGRP (Lv et al., 2022). CGRP plays important roles in migraine pathophysiology, inflammatory response, and blood pressure (Mehkri et al., 2022). At present, good progress has been made in the drug research of GCRP and CGRPR, and four related monoclonal antibodies have been developed (Deganutti et al., 2021). In addition, GCRP plays an important role in the cochlea. GCRP is expressed in the lateral olivocochlear (LOC), medial olivocochlear (MOC) efferent neurons and type II SGNs (SGN IIs) and up-regulates excitatory of auditory nerve (AN) activity (Schrott-Fischer et al., 2007; Vyas et al., 2019; Le Prell et al., 2021). In α*CGRP* knockout mice, ABR thresholds were reduced and hearing impairment was presented (Maison et al., 2003). Additionally, the CGRPR complex in the cochlea exhibits maturation during the first 3 months, which corresponds to an increase in cochlear nerve activity (Dickerson et al., 2016). However, research on CGRPR in the cochlea is relatively lagging, particularly the location and specific function of CGRPR in the cochlea are currently unknown.

## Roles of class B2 G protein-coupled receptors in cochlea

### Adhesion G protein-coupled receptor C

Cadherin EGF LAG Seven-pass G-type Receptor 1 (CELSR1), also named as Adhesion G-protein Receptor C1 (ADGRC1), is mainly distributed in the nervous system. In humans and mice, mutations in *CELSR1* strongly affect neural tube development (Ravni et al., 2009; Allache et al., 2012).

CELSR1 is expressed in both inner ear HCs and SCs, and is considered as a key Planar cell polarity (PCP) protein in the cochlea to be involved in cellular communication and coordination between HCs and SCs (Shima et al., 2002; Curtin et al., 2003; Davies et al., 2005). In two *CELSR1* mutant mice (*Scy* and *Crsh*), the OHCs were massively misoriented, most severely at the apex of the cochlea (Curtin et al., 2003). Interestingly, no significant auditory HC dislocation and hearing impairment were observed in *CELSR1* knockout mice but not in mutant strains, which may be due to compensatory effects from other *CELSR* genes (e.g., *CELSR2, 3*) (Tissir and Goffinet, 2006; Duncan et al., 2017). In addition, there are some PCP proteins in cochlear HCs, but whether and how CELSR1 cooperates with other PCP proteins to effect on the plane polarity of HCs, which is largely unknown and needs to further study.

### Adhesion G protein-coupled receptor V

Very large G protein-coupled receptor 1 (VLGR1), known as MASS1, Adhesion G-protein Receptor V1 (ADGRV1), Neurepin and G protein-coupled receptor 98 (GPR98), is to date the largest known protein in GPCR super-families including about 6,300 amino acid residues (Sun et al., 2013). Several *VLGR1* mutations have been reported to cause Usher syndrome type IIC (USH2C) in humans, a genetically heterogeneous autosomal recessive disorder characterized by hearing impairment and epileptic seizures (Kimberling et al., 1995; Ebermann et al., 2007; Bonnet and El-Amraoui, 2012).

In the inner ear, VLGR1 is expressed in the ankle region of HCs stereocilia, which can form the ankle-link complex with Usherin, Vezatin, and Whirlin (Michalski et al., 2007). VLGR1 was identified to form a complex with Clarin-1, CDH23, and PCDH15 at the ribbon synapses of HCs (Zallocchi et al., 2012b), and can also interact with various proteins including Harmony (Verpy et al., 2000), PDZ7 (Colcombet-Cazenave et al., 2022), MyosinVIIa (Michalski et al., 2007), and SNAP25 (Zallocchi et al., 2012a). In various *VLGR1* mutant or knockout mouse models, the stereociliary development of auditory HCs is impaired, the ankle-links are absent, and hearing impairment of varying degrees occurs (Skradski et al., 2001; McMillan and White, 2004; Yagi et al., 2005). Collectively, VLGR1 can carry out the stereociliary development and hearing function.

### Adhesion G protein-coupled receptor A

G protein-coupled receptor 125 (GPR125) is also named as adhesion G protein-coupled receptor A3 (ADGRA3), and involve in regulating planar cell polarity signaling (Li et al., 2013). GPR125 is widely expressed in the cochlea, especially in OHCs, SGNs, and interdental cells (ICs) (Sun et al., 2021). However, in GP125-deficient mice, various types of cells developed normally, and hearing function was not impaired (Sun et al., 2021), implying that GPR125 may not regulate the planar cell polarity in the cochlea.

## Roles of class C G protein-coupled receptors in cochlea

### Metabotropic glutamate receptors

The metabotropic glutamate receptor (mGluR) family includes eight known subtypes (mGluR1∼8) that are subdivided into three groups (group I-III) (Niswender and Conn, 2010; Reiner and Levitz, 2018; Ge and Wang, 2022). In general, group I (mGluR1 and mGluR5) mainly positively regulate the activity of glutamatergic synapses. In contrast, both group II (mGluR2 and mGluR3) and III mGluR (mGluR4, mGluR6∼8) function in limiting the release of neurotransmitters. In addition, most mGluRs can be alternatively spliced at the intracellular C-terminus to generate isoforms such as mGluR7a and mGluR7b, and then form homo- and heterodimers for dynamic regulation (Seebahn et al., 2011; Habrian et al., 2019).

Among all mGluRs, mGluR1 is present both in the SGNs and HCs (Ye et al., 2017). mGluR4, mGluR7a, mGluR7b, and mGluR8b were found at the pre-synaptic ribbons of IHCs, while mGluR2 is localized at post-synaptic type I SGNs (SGN Is) and efferent lateral olivocochlear GABAergic fibers (Doleviczenyi et al., 2005; Klotz et al., 2019; Klotz and Enz, 2021). Moreover, mGluR7 and mGluR8 can be detected at the OHCs (Friedman et al., 2009; Girotto et al., 2014). Especially, mGluR1 can enhance efferent inhibition of developing IHCs and promote excitatory neurotransmission in SGN Is (Peng et al., 2004; Ye et al., 2017). In contrast, mGluR2 can protect cochlea from damage by inhibiting efferent dopamine release onto IHCs (Doleviczenyi et al., 2005). Interestingly, mGluR7 is also associated with ARHL and NIHL in humans (Friedman et al., 2009; Newman et al., 2012; Chang et al., 2018; Yu et al., 2018; Matyas et al., 2019), and mGluR7 knockout mice exhibited hearing deficits (Fisher et al., 2020). These studies suggest that mGluRs, especially group II and III mGluRs, can play a key role in preventing excitotoxicity induced by excessive glutamate release from IHCs. However, mGluR4 and mGluR8b, which co-localize with the mGluR7, warrant further investigation of their specific functions in IHCs. In addition, whether mGluR4, mGluR7a, mGluR7b, and mGluR8b can form different homologous and/or heterodimeric receptors to execute diverse roles in IHCs deserves further investigation.

### γ-Aminobutyric acid B receptors

The γ-aminobutyric acid receptor type B (GABA B receptor), as a metabotropic receptor, can be activated by γ-aminobutyric acid (GABA) and mediate long-term, slow signaling responses mainly in the form of heterodimers (Mao et al., 2020; Fritzius et al., 2022; Vlachou, 2022). GABAB receptors are composed of two distinct subunits, GB1 and GB2. Due to the alternative splicing of GB1 subunit mRNA, 14 different GB1 isoforms can be generated, of which GB1a and GB1b are most widely studied (Bowery and Enna, 2000; Shaye et al., 2021; Shen et al., 2021). What’s more, GABA B receptors were found to be localized at all mature or nascent cochlear SGNs, including SGN I and SGN II, but not in HCs (Lin et al., 2000; Reijntjes and Pyott, 2016). GABA B receptors affect OHC function in the cochlea. In mice knocked out of GABA B1, hearing thresholds increased by about 10 dB (Maison et al., 2009). In addition, GABA B(1a,2) on the SGN modulates the strength of the SGN-HC synapse by inhibiting the release of acetylcholine (Ach) following GABA activation (Wedemeyer et al., 2013). In contrast to the few studies that work in the cochlea, research on GABA B receptors in the auditory domain is currently focused on the cochlear nuclear complex (CNC) of the brain (Kou et al., 2013; Qu et al., 2015). Whether there is a potential correlation between the GABA B receptors of the cochlea and CNC is worthy of follow-up study.

### Calcium-sensing receptors

The calcium-sensing receptor (CaSR) works as a key regulator by sensing extracellular Ca^2+^ fluctuations to affect downstream intracellular signaling pathways (Tuffour et al., 2021). Thus, CaSR plays a key role in maintaining cellular Ca^2+^ homeostasis. In cochlea, Ca^2+^ homeostasis is also essential for acoustic transduction and proper development of cochlea, including synaptic transmission, mechanoelectrical transduction and the network of SCs (Ceriani and Mammano, 2012; Sirko et al., 2019). CaSR expression is detected in fibrocytes of the spiral ligament and spiral limbus, smooth muscle cells (SMCs) of the spiral modiolar arteries and epithelia of the osseous spiral lamina (Wonneberger et al., 2000; Minakata et al., 2019). Only one work has reported the role of CaSR in cochlear fibrocytes, where CaSR can regulate Ca^2+^ concentration (Minakata et al., 2019). When the CaSR inhibitor (NPS2143 and Calhex231) was used, the hearing threshold increased by 20–30 dB, indicating that the Ca^2+^ signal mediated by CaSR is required for hearing. Therefore, the study of the complete regulatory pathway of CaSR to maintain cochlear Ca^2+^ homeostasis will help to treat hearing loss.

### Class C orphans receptors

GPR156, as an orphan GPCR of class C, has a significant sequence homology with GABA B receptor (Calver et al., 2003), and has a high G_*i/o*_ constitutive activity (Watkins and Orlandi, 2021). GPR156 is currently only reported as a key regulator of orientation in sensory HCs (Kindt et al., 2021). GPR156 is expressed in all HCs of the cochlea, and knockout of GPR156 causes hearing loss but not HC death (Kindt et al., 2021). GPR156 distribution can be polarized by the transcription factor EMX2, which is then signaled by Gα_*i*_ to trigger a 180° reversal of HC orientation (Kindt et al., 2021). The EMX2 > GPR156 > Gα_*i*_ signaling cascade is therefore required for HC orientation (especially OHC1 and OHC2) and hearing function. In this signaling cascade, how EMX2 affects GPR156 and whether there are agonists combined with GPR156 to participate in the reversal of HCs worth further research.

## Roles of class F G protein-coupled receptors in cochlea

### Frizzled receptors

Class F of GPCR or frizzled GPCR family includes ten Frizzleds (FZD1-10) and Smoothened (SMO), all of them have this cysteine-rich domain (CRD) in their extracellular region (Schulte and Wright, 2018; Zhang X. et al., 2018; Kozielewicz et al., 2020; Schulte and Kozielewicz, 2020). These receptors play key roles in embryonic development, cellular polarity, proliferation, differentiation, and maintenance of stem cells.

The 10 FZDs coordinate the Wnt signaling in two ways: through disheveled (DVL1-3)–dependent pathway (Clevers and Nusse, 2012; Grainger and Willert, 2018) and through heterotrimeric G-protein-mediated pathway (Dijksterhuis et al., 2014; Kilander et al., 2014). In addition to FZD5 and FZD8, the other eight FZDs have been reported to couple to various types of G proteins (Schulte and Wright, 2018). Most of these *FZD*s were detected by RT-PCR in the rat cochlea (Daudet et al., 2002) and RNA *in situ* hybridization in the chicken cochlea (Sienknecht and Fekete, 2008). In the mammalian cochlea, *FZD1* and *FZD2* were both expressed at lower levels in sensory HCs, but at higher levels in SCs (Yu et al., 2010). And the expression of *FZD4* can be detected in auditory and vestibular HCs (Wang et al., 2001). Furthermore, both FZD3 and FZD6 are expressed in cochlear SCs (Ghimire and Deans, 2019) and the medial side of HCs (Montcouquiol et al., 2006; Chang et al., 2016), but FZD3 is expressed in SGNs (Duncan et al., 2019; Stoner et al., 2021). While the expression of FZD9 can be detected in early cochlear inner phalangeal cells (IPhCs), IBCs, and the third-row DCs (Zhang et al., 2019).

Among the 10 FZDs, FZD 2, 3, 4, 6, and 9 play different important roles in the cochlea. Knockout of *FZD2* in the mouse cochlea results in defects in OHC number and orientation, which are most severe in the apical region. In addition, a recent study identified *FZD2* as a signature gene in one of three distinct SGN I populations by using single-cell transcriptome (Grandi et al., 2020). In the mouse cochlea, planar polarity is guided by the PCP proteins FZD3 and FZD6. However, FZD3 and FZD6 are functionally redundant, and the orientation of HCs is severely affected only in *FZD3/6* double knockout mice (Wang et al., 2006). Moreover, FZD3 and FZD6 play a key role in guiding SGN II peripheral axons turning (Ghimire and Deans, 2019). A recent study uncovered SPAG6 as a regulator of FZD6, because FZD6 lost its normal polarized distribution in *SPAG6*^–/–^ mice (Li et al., 2021). Furthermore, a novel non-canonical Wnt pathway was identified in cochlear HCs that signals through PI3K, Rac1 and Gsk3β to regulate the PCP pathway by promoting the junctional localization of core PCP proteins such as FZD6 (Landin Malt et al., 2021). Compared with the above-mentioned FZDs, FZD4 does not affect HC survival but only affects HC function, so only the late onset hearing loss can be found in *FZD4*^–/–^ mice (Wang et al., 2001). The main role of FZD9 in cochlea is HC regeneration, and *FZD9*^+^ cells have strong ability of proliferation, differentiation and HC generation. It still has HC-generating capacity at 6 days after treatment *in vivo* lineage tracing, especially it can act as an effective marker for HC progenitors (Zhang et al., 2019). Therefore, FZD9 has clinical translational value for the regeneration of HCs.

SMO mediates the Hedgehog (Hh) signaling pathway, activated SMO lead Gli to translocate into the nucleus to activate target genes, in which SMO can couple to G_*i/o*_ and G_12/13_ (Gorojankina, 2016; Qi et al., 2019; Okashah et al., 2020). In the auditory field, SMO is found to participate in cochlear development, HC differentiation and hearing function. In mouse embryos carried inner ear conditional knockout of *Smoothened* (*SMO^ecko^*), the cochlea exhibited hypoplasia, in which the cochlear duct and saccule were completely absent in *SMO^ecko^* embryos (Brown and Epstein, 2011; Muthu et al., 2019). What’s more, in *SMO^cko^* mouse (similar to *SMO^ecko^* mouse) early cochlea, apex HCs preferentially accelerate differentiation (Tateya et al., 2013). Although *SMO^cko^* mice survive after birth, HCs in the apical region appear disorganized and reduced in number, causing hearing loss which predominantly at low frequencies. And *SMO^ecko^* has also been used to identify key genes that are activated and repressed by Shh signaling in the cochlea during the initial stages of growth (Muthu et al., 2019). In addition, SMO may be associated with otosclerosis (Brown and Epstein, 2011) and cochlear neural stem cell (NSC) transplantation (Huang et al., 2018). The use of taurine during transplantation can up-regulate Hh pathway proteins such as SMO, thereby stimulating the cell proliferation and differentiation of NSCs into SGNs. These results suggest that SMO has potential applications in the treatment of hearing impairment and cochlear NSC transplantation.

## Emerging G protein-coupled receptor-based treatment

### G protein-coupled receptor-based drug development

**FIGURE 2:**
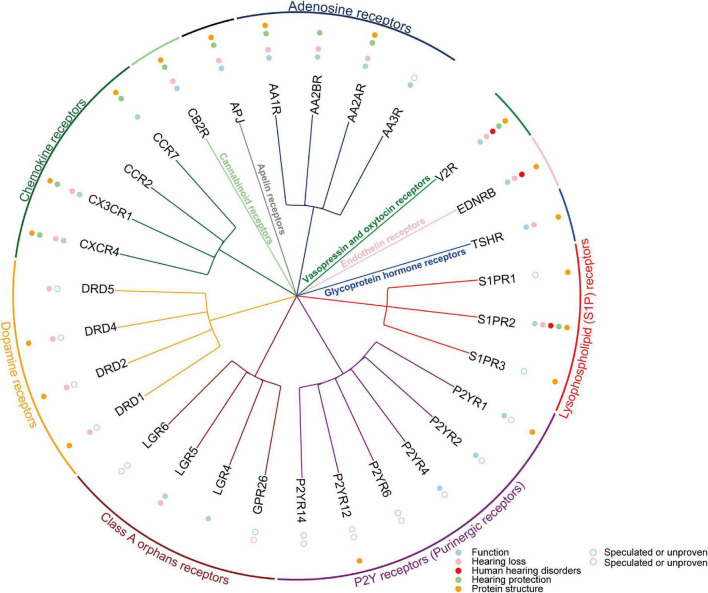
The summary of reported 30 Class A GPCRs in cochlea. The blue circle indicates that GPCR are functional in the cochlea. Pink circle indicates GPCR associated with hearing loss. Red circle indicates GPCR associated with human hearing disorders. Green circle indicates GPCR involved in hearing protection. The orange circle indicates that the GPCR has resolved the protein structure. Open circles indicate conjectures or confirmed conclusions.

**FIGURE 3:**
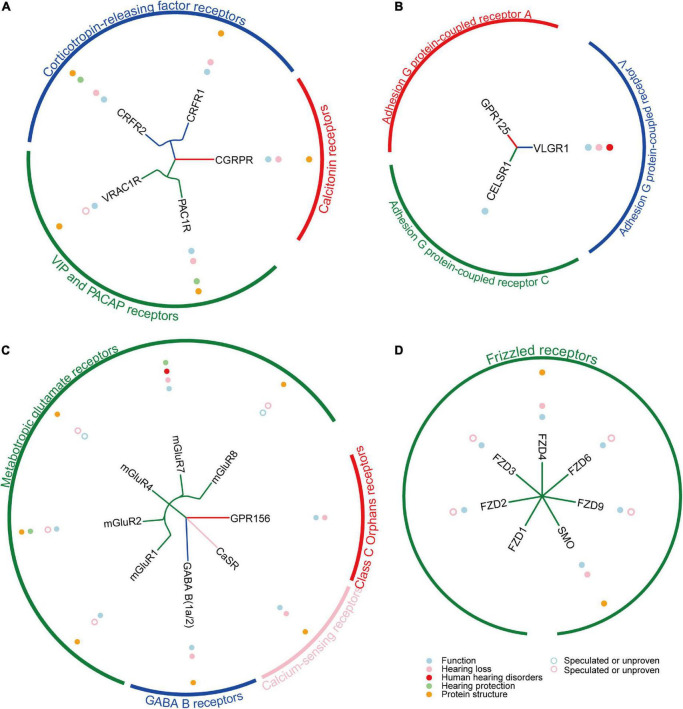
The summary of reported Class B1/B2/C/F GPCRs in cochlea. **(A)** The summary of reported 5 Class B1 GPCRs in cochlea. **(B)** The summary of reported 3 Class B2 GPCRs in cochlea. **(C)** The summary of reported 8 Class C GPCRs in cochlea. **(D)** The summary of reported 7 Class F GPCRs in cochlea.

G protein-coupled receptors are keeping great advantages as drug targets, thanks to the rapid development of single-particle cryo-electron microscopy (cryo-EM) technology and artificial intelligence (AI) technology in recent years. Since the first use of cryo-EM to resolve the complex structure of GPCR and G protein in 2017, the number of GPCR structures resolved every year has grown exponentially (Liang et al., 2017; Kooistra et al., 2021). Compared with previous X-ray crystallography studies required higher thermal stability, cryo-EM can obtain different conformations of stable GPCRs and structures of complexes with G proteins that are closer to the native state, which greatly improves the efficiency of ligand screening or drug design (Renaud et al., 2018). So far, the structures of 30 GPCRs functioning in the cochlea have been successfully resolved (Figures 2, 3; Supplementary Table 1). In recent years, the application of AI in structural biology has also greatly promoted the elucidation of GPCRs and the corresponding drug design. For example, AlphaFold2 and RosettaFold are the most typical applications at present (Baek et al., 2021; Jumper et al., 2021) with high accuracy, high speed, and convenience for GPCR structure-oriented drug design. In addition, according to statistics, GPCRs remain one of the most important drug targets (Hauser et al., 2017).

There are many types of GPCRs targeting drugs in clinical trials, including peptides, monoclonal antibodies, recombinant proteins, small molecules, and nanobodies (Saikia et al., 2019). According to the mode of action, it can be divided into agonists, antagonists, positive allosteric modulators (PAM), negative allosteric modulators (NAM) and so on (Hauser et al., 2017; Odoemelam et al., 2020). When a ligand binds to a GPCR, the receptor undergoes a conformational change in which agonists can activate and antagonists can inhibit signal transduction pathways. In contrast to orthosteric ligands such as agonists and antagonists, allosteric modulators (PAM or NAM) as promising therapeutic agents can infiltrate into a pocket that is different in space than the orthotopic site, and modulate signaling only in the presence of the natural ligand to prevent adverse side effects (Massink et al., 2020; Yang et al., 2022).

In cochlear research and treatment, GPCR-targeted drugs have also been used to some extent. For example, S1PR2 agonist (CYM-5478) (Wang et al., 2020), CaSR inhibitors (NPS2143 and Calhex231) (Minakata et al., 2019), V2R antagonist (OPC-41061/Tolvaptan) (Egami et al., 2016), CB2R agonist (JWH105) (Vlajkovic et al., 2006; Dhukhwa et al., 2021), AA1R agonists (R-PIA and ADAC) (Vlajkovic et al., 2010; Kaur et al., 2016), AA2AR antagonist (istradefylline) (Han et al., 2019; Manalo et al., 2020; Shin et al., 2021) all have a therapeutic and protective effect on the cochlea. Among these GPCR-targeted drugs, V2R antagonist (OPC-41061/Tolvaptan) and AA2AR antagonist (istradefylline) have been approved by the US Food and Drug Administration (FDA) (NDA022075, NDA022275). V2R antagonist (OPC-41061/Tolvaptan) is approved for the treatment of autosomal dominant polycystic kidney disease, fibrosis, hyponatremia, heart failure, and the syndrome of dysregulated antidiuretic hormone secretion in humans (Cao et al., 2022; Martin-Grace et al., 2022). AA2AR antagonist (istradefylline) is widely used to treat Parkinson’s disease in humans (Merighi et al., 2022). However, many GPCRs extensively developed in the cochlea, such as CRFR1 (Graham and Vetter, 2011), CRFR2 (Graham et al., 2010), and CGRPR (Maison et al., 2003) mentioned above, also have great potential in the future of cochlear therapy to treat or prevent of noise- and pharmaceutical-induced auditory toxicity (Lowery and Thiele, 2010; Hauser et al., 2017; Deganutti et al., 2021). Therefore, besides existing drugs targeting GPCRs that can be further tried to be applied to the treatment of the cochlea, more structures of potential GPCRs targets are helpful to design drugs in cochlear therapy.

### G protein-coupled receptor-based gene therapy

The use of drugs generally only works when the target protein exists and expresses. Considering that one out of every 1,000 births in the world is hereditary deafness (Ajay et al., 2022), and mutations or deletions of 124 genes have been found to cause hearing loss,^2^ so these hereditary hearing impairments are difficult to treat with drugs. Therefore, gene therapy provides a therapeutic direction for hearing loss caused by gene mutation or deletion (Lee et al., 2020; Maguire and Corey, 2020). Briefly, the introducing a normal gene into the target cell through a delivery vector to replace or enhance the defective gene, can restore a normal level to avoid loss of function. In the field of cochlear therapy, gene therapy was first used to rescue hearing loss in the *VGLUT3* knockout mouse, a model of congenital deafness, as early as 2012 (Akil et al., 2012). Subsequently, many deafness caused by gene mutation or deletion were studied by similar gene therapy approaches, including *TMC1* (Nist-Lund et al., 2019), *GJB2* (Iizuka et al., 2015), *USH1C* (Pan et al., 2017), and so on.

In recent years, the CRISPR (clustered regularly interspaced short palindromic repeats) based gene editing methods have opened up new avenues for gene therapy in the field of hearing (Zuris et al., 2015). It can target gene disruption or repair mutations to restore gene function, no need to consider how to produce adequate levels of exogenous transgene expression. At present, the exploration of gene editing methods for cochlear gene therapy is mainly based on CRISPR-Cas9 and CRISPR-Cas13 (Geleoc and El-Amraoui, 2020; Botto et al., 2021). The CRISPR-Cas9 system edits DNA, and the cure may be permanent after the correction of disease-causing mutation *in vivo*. It has been studied in a series of hearing treatments, for example, the dominant mutation of the *TMC1* gene in a Beethoven mouse model of hearing loss has been successfully corrected. Hearing was significantly restored in the treated mice, and this effect was stable for up to a year (Gao et al., 2018; Gyorgy et al., 2019). Unlike the CRISPR-Cas9 system targeting DNA, CRISPR-Cas13 system edits disease-associated RNA transcripts, which is transient and potentially reversible, thus also offering improved safety. Two recent studies have revealed the potential of the CRISPR-Cas13 system in gene therapy for the repair of hearing loss caused by mutations, including CRISPR-Cas13X (Xiao et al., 2022) and CRISPR-CasRx (Guo et al., 2022).

The good news is that the first gene therapy for a disease caused by a specific genetic mutation has been approved by the FDA at 2017 (Maguire et al., 2021), supporting the huge clinical potential of gene therapy. In the field of GPCR-related gene therapy, considerable progress has been made in the study of rhodopsin (RHO) in the retina (Athanasiou et al., 2018). Multiple works rescue retinal degeneration in *RHO* mutant mice for up to 6–9 months by supplementing exogenous *RHO* (O’Reilly et al., 2007; Chadderton et al., 2009; Mao et al., 2011). In addition, there is some work to correct *RHO* mutations by CRISPR/Cas9 gene editing for the treatment of inherited retinal degeneration (Bakondi et al., 2016; Burnight et al., 2017). Among the nine genes with mutations or deletions of GPCR-encoding genes that cause hearing loss, including *GABA B1* (Maison et al., 2009), *GPR156* (Kindt et al., 2021), *mGluR7* (Friedman et al., 2009), *VLGR1* (Kimberling et al., 1995), *PAC1R* (Ruel et al., 2021), *EDNRB* (Huang et al., 2021), *S1PR2* (Santos-Cortez et al., 2016), *TSHR* (Li et al., 1999), and *CRFR1* (Graham and Vetter, 2011), five of them *mGluR7* (Friedman et al., 2009), *VLGR1* (Kimberling et al., 1995), *EDNRB* (Huang et al., 2021), *V2R* (Zou et al., 2019; Wang et al., 2022), and *S1PR2* (Santos-Cortez et al., 2016) are directly related to human deafness, which are especially worth developing for gene therapy.

## Conclusion

In this review, we mainly summarize the expression of five subfamilies of GPCRs in the cochlea, their functions, their relationship with hearing loss, and their potential therapeutic directions (Tables 1–5). A total of 53 GPCRs have been reported to be expressed in the cochlea, of which 38 have been shown to function in the cochlea (Figures 2, 3; Supplementary Table 1). Most importantly, 27 GPCRs were found to be associated with hearing loss, 5 of which were directly associated with human hearing disorders (VLGR1, mGluR7, V2R, EDNRB, and S1PR2). In addition, 13 GPCRs (CXCR4, CX3CR1, CCR2, CCR7, CB2R, APJ, AA1R, AA2AR, AA2BR, PAC1R, CRFR2, mGluR7, and S1PR2) were confirmed to play a hearing protective role in noise and ototoxicity. We also prospect the GPCR-targeted drug development and gene therapies in the future. In conclusion, GPCRs have great potential in the treatment of hearing loss, so more GPCR functions in the cochlea, more GPCRs related to hearing loss, and more GPCR-based treatment regimens remain to be further explored.

**TABLE 2:** Class B1 GPCRs relevant to cochlea.

GPCR family	Subtypes	Roles in cochlea	Localization	Genetic modulation	References
**Class B1 GPCRs**					
VIP and PACAP receptors	VPAC1R	Possibly acting as neurotransmitters	SGN	-	Feng and Liu, 2015
	PAC1R	Protection of NIHL	HC, SC, SGN, ANF, ENF, SV	*PAC1R^–/–^* mice	Ruel et al., 2021
				*TgHPAC1R* mice	
Corticotropin-releasing factor receptors	CRFR1	Maintaining normal auditory function	ISC, HSC, DC, BC	*CRFR1^–/–^* mice	Graham and Vetter, 2011
		Involved IHC and HC innervation development		*CRFR1-GFP* mice	
	CRFR2	Constitutively modulates hearing sensitivity	ISC, HSC, DC, IBC, SGNs, CC, BoC	*CRFR2^–/–^* mice	Graham et al., 2010
		Protection of NIHL			
**Calcitonin receptors**	CGRPR	Associated with increased cochlear nerve activity	-	-	Dickerson et al., 2016

SGN, Spiral Ganglion Neuron; HC, hair cell; SC, supporting cell; ANF, afferent nerve fiber; ENF, efferent nerve fiber; SV, Stria Vascularis; ISC, inner sulcus cell; HSC, Hensen cell; DC, Deiters’ cell; BC, border cell; IBC, inner border cell; CC, Claudius cell; BoC, Boettcher cell.

**TABLE 3:** Class B2 GPCRs relevant to cochlea.

GPCR family	Subtypes	Roles in cochlea	Localization	Genetic modulation	References
**Class B2 GPCRs**					
Adhesion G protein-coupled receptor C	CELSR1	Guide OHC orientation	HC, SC	*CELSR1^–/–^* mice	Duncan et al., 2017
				*CELSR1^*Crsh*/Crsh^* mouse	Curtin et al., 2003
				*CELSR1^*Scy*/Scy^* mice	
Adhesion G protein-coupled receptor V	VLGR1	Involved in the stereociliary development of hair cells	HC	*VLGR1^–/–^* mice	Yagi et al., 2005
		Cause Usher syndrome type IIC (USH2C) in humans		*VLGR1/delTM7* mice	McMillan and White, 2004
				*VLGR1^*BUB*/BnJ^* mice	Skradski et al., 2001
				*VLGR1^Frings^* mice	
Adhesion G protein-coupled receptor A	GPR125	No effect on cochlear development and hearing	OHC, SGNS, IC	*GPR125^–/–^* mice	Sun et al., 2021
				*Gpr125^*lacZ*/lacZ^* mice	

HC, hair cell; SC, supporting cell; OHC, outer hair cell; SGN, Spiral Ganglion Neuron; IC, interdental cell.

**TABLE 4:** Class C GPCRs relevant to cochlea.

GPCR family	Subtypes	Roles in cochlea	Localization	Genetic modulation	References
**Class C GPCRs**					
Metabotropic glutamate receptors	mGluR1	Enhance efferent inhibition of IHCs	SGN, HC	-	Ye et al., 2017
		Promote excitatory neurotransmission in SGN I			Peng et al., 2004
	mGluR2	Inhibit efferent dopamine release onto IHCs	SGN I, efferent lateral olivocochlear GABAergic fiber	-	Doleviczenyi et al., 2005
	mGluR4	-	IHC	-	Klotz et al., 2019
	mGluR7	Associated with ARHL and NIHL	HC	*mGluR7^–/–^* mice	Chang et al., 2018
		Knockout results in hearing deficits			Fisher et al., 2020
	mGluR8	-	HC	-	Klotz et al., 2019
GABA B receptors	GABA B(1a,2)	Affect OHC function	SGN	*GABA B1-GFP reporter* mouse	Maison et al., 2009
		Modulate the strength of the SGN-HC synapse		*GABA B1a^–/–^* mice	Wedemeyer et al., 2013
				*GABA B1b^–/–^* mice	
Calcium-sensing receptors	CaSR	Maintain cochlear Ca^2+^ homeostasis	Fibrocytes of the spiral ligament and spiral limbus, SMC of the spiral modiolar arteries and epithelia of the osseous spiral lamina	-	Minakata et al., 2019
Class C Orphans receptors	GPR156	EMX2 > GPR156 > Gα_i_ signaling cascade is required for HC orientation	HC	*GPR156^–/–^* mice	Kindt et al., 2021
				*GPR156^*exon*2^* zebrafish	
				*GPR156^*sa*34566^* zebrafish	

SGN, Spiral Ganglion Neuron; HC, hair cell; IHC, inner hair cell; SMC, smooth muscle cell.

**TABLE 5:** Class F GPCRs relevant to cochlea.

GPCR family	Subtypes	Roles in cochlea	Localization	Genetic modulation	References
**Class F GPCRs**					
Frizzled receptors	FZD1	-	HC, SC	*FZD1*^–/–^ mice	Yu et al., 2010
	FZD2	Guide OHC orientation	HC, SC, SGN I	*FZD2*^–/–^ mice	Yu et al., 2010
		A marker as one distinct type I SGN			Grandi et al., 2020
	FZD3	Guide planar polarity of HC with FZD6	HC, SC, SGN	*ATOH1*-Cre: *FZD3*^–/–^ mice	Stoner et al., 2021
		Guide Type II SGN peripheral axons turning with FZD6		*NEUROD1*-cre: *FZD3*^–/–^ mice	Wang et al., 2006
				*FZD3*^–/–^ mice	Ghimire and Deans, 2019
				*FZD3*^–/–^; *FZD6*^–/–^ mice	
	FZD4	Knockout results in the late onset hearing loss	HC	*FZD4*^–/–^ mice	Wang et al., 2001
	FZD6	Guide planar polarity of HC with FZD3	HC, SC	*FZD6*^–/–^ mice	Wang et al., 2006
		Guide Type II SGN peripheral axons turning with FZD3		*FZD3*^–/–^; *FZD6*^–/–^ mice	Ghimire and Deans, 2019
	FZD9	Promote hair cell regeneration	IPhC, IBC, DC	*FZD9-CreER; ROSA26-tdTomato* Mice	Zhang et al., 2019
	SMO	Affect cochlear development: cochlear duct and saccule were absent when knockout	HC, NSC	*FOXG1-Cre; SMO^*loxp*/–^* (*SMO^ecko^*) mice	Muthu et al., 2019
		Affect hair cell differentiation: apical HCs appear disorganized and reduced when knockout		*EMX2-Cre; SMO^–/–^* (*SMO^cko^*) mice	Brown and Epstein, 2011
		Affect hearing function: hearing loss at low frequencies when knockout			Tateya et al., 2013
		Promote cochlear NSC transplantation			Huang et al., 2018

HC, hair cell; SC, supporting cell; SGN, Spiral Ganglion Neuron; IPhC, inner phalangeal cell; IBC, inner border cell; DC, Deiters’ cell; NSC, neural stem cell.

## Author contributions

YY, JF, and RC conceived this review. XM and JG wrote the manuscript. YF, CS, PJ, YZ, LZ, YY, JF, and RC revised the manuscript. All authors have read and approved the final manuscript.

## Footnotes

1
www.who.int/publications-detail-redirect/world-report-on-hearing

2
https://hereditaryhearingloss.org/
